# Discrepancies between caliper and portable A-mode ultrasound estimates of subcutaneous fat in older adults

**DOI:** 10.1038/s41430-026-01756-9

**Published:** 2026-04-29

**Authors:** Lara Vilar Fernandes, Gabriela Benatti de Oliveira, Rui Valdiviesso, Teresa F. Amaral, Ligiana Pires Corona

**Affiliations:** 1 UNICAMP, FCA, School of Applied Sciences, University of Campinas, Limeira, Brazil; 2 UNICAMP, FCM, School of Medical Sciences, University of Campinas, Campinas, Brazil; 3 FCNAUP, Faculty of Nutrition and Food Sciences of the University of Porto, Porto, Portugal; 4 RISE-Health, Faculty of Nutrition and Food Sciences of the University of Porto, Porto, Portugal; 5 IUCS, University Institute of Health Sciences-CESPU, Gandra, Portugal; 6 LAETA/INEGI, Associate Laboratory of Energy, Transports and Aeronautics, Institute of Science and Innovation in Mechanical and Industrial Engineering, Porto, Portugal

**Keywords:** Geriatrics, Nutrition

## Abstract

It has been proposed that subcutaneous fat thickness measured by ultrasound corresponds to half of the skinfold thickness measured with calipers at the same anatomical site. This study compared portable A-mode ultrasound and caliper skinfold assessments in older adults. In a cross-sectional sample of 146 participants, fat thickness was measured at the biceps brachii, triceps brachii, subscapular, and suprailiac sites using a BodyMetrix® BX-2000 ultrasound device and a LipoWise PRO digital caliper. Agreement between methods varied markedly by site: Lin’s concordance correlation coefficients were 0.21 (triceps), 0.02 (subscapular), and 0.33 (biceps, suprailiac), indicating poor to moderate concordance. The Bradley–Blackwood test showed differences between methods (*p* < 0.001). Skinfold/ultrasound mean ratios were 1.50 (triceps), 1.20 (biceps), 1.50 (suprailiac), and 2.90 (subscapular). Caliper skinfold measurements consistently overestimate subcutaneous fat thickness versus ultrasound, especially at the subscapular site, limiting interchangeability and potentially compromising clinical assessment accuracy in older adult populations.

## Introduction

The increasing prevalence of obesity in older adults is associated with metabolic dysfunction, sarcopenic obesity, and higher risks of cardiovascular disease, diabetes, and frailty, reinforcing the importance of body composition assessment in this population [1]. Skinfold thickness provides a practical, non-invasive, and low-cost method for estimating body composition, though its accuracy may be affected by age-related changes in skin elasticity, hydration, and fat distribution [2, 3]. Ultrasound has emerged as an alternative technique for measuring subcutaneous fat thickness, offering greater potential for standardisation, portability, and repeated assessments without radiation exposure [4]. A-mode ultrasound, although lower in spatial resolution than B-mode, provides a portable and low-cost alternative for subcutaneous fat assessment with evidence of comparable validity [4]. Nevertheless, additional studies are needed to confirm its validity and reliability in diverse older populations.

Although studies have compared skinfold thickness measured by calipers with subcutaneous adipose tissue thickness assessed by ultrasound, most of the available evidence comes from younger or athletic populations [5–7]. Agreement between skinfold and ultrasound measures of subcutaneous fat in older adults remains limited, particularly with portable A-mode devices. Age-related tissue changes may alter measurement relationships, highlighting the need for population-specific validation to ensure accurate assessment and clinical interpretation.

Therefore, this study aims to compare subcutaneous fat estimates obtained by portable A-mode ultrasound and skinfold caliper in older adults.

## Materials and methods

This cross-sectional study included 150 community-dwelling older adults (≥60 years) from Campinas, Brazil, recruited from the UNICAMP UniversIDADE Program, other UNICAMP community members, and the Geriatric Outpatient Clinic of the Clinics Hospital at UNICAMP. The study was approved by the Ethics Committee of the State University of Campinas (CAAE: 51443321.0.0000.5404), and all participants provided written informed consent.

Inclusion criteria were age ≥60 years, residence in Campinas or surrounding areas, and adequate neurological and cognitive conditions to answer the questionnaires. Exclusion criteria included conditions affecting body composition, such as chronic obstructive pulmonary disease, dialysis-dependent kidney disease, Parkinson’s disease, heart failure, or human immunodeficiency virus–positive status, as well as home care or chemotherapy.

Subcutaneous fat thickness was measured using the portable A-mode ultrasound BodyMetrix® BX-2000 (IntelaMetrix, Inc., Livermore, CA, USA; version 2.1.0.9045) by two trained evaluators, following the manufacturer’s guidelines and prior practice on volunteers to ensure proficiency and image quality. Subcutaneous fat thickness was measured at the triceps brachii, biceps brachii, subscapular, and suprailiac sites using the scan mode, following the manufacturer’s guidelines. All procedures related to participant positioning (standing), use of ultrasound gel, probe placement (perpendicular to the skin), and probe pressure (sliding slowly to reduce pressure) followed the manufacturer’s instructions. The ultrasound site was marked once, and both raters used the same skin marking for all measurements. Two trained evaluators obtained three measurements per site; the two clearest images from each were averaged for analysis, ensuring high ICC > 0.90 [8, 9]. Reliability was based on a two-way mixed-effects model. The arithmetic mean of the four ultrasound-derived subcutaneous fat thickness values (to clearest images of each evaluator) was used for statistical analyses. Markings were placed at the midpoint of the image (1/2).

Skinfold thickness was measured at the triceps brachialis, biceps brachialis, subscapular, and suprailiac sites using the validated Lipowise PRO digital caliper (Wisify, Porto, Portugal) [10]. A single experienced evaluator performed triplicate measurements at each site, and the mean value was used for analysis. Systematic bias related to measurement sequence was unlikely, with appropriate intervals between measurements.

Quantitative variables were expressed as mean ± SD or median (IQR), depending on normality assessed by the Shapiro–Wilk test. Sex differences were evaluated using independent t-tests or Mann–Whitney U tests. Comparisons between skinfold and ultrasound measurements were performed with paired t-tests or Wilcoxon signed-rank tests, as appropriate. Agreement between skinfold and ultrasound measurements was assessed using Lin’s concordance correlation coefficient (CCC) and Bland–Altman analysis, with 95% limits of agreement. Bias was evaluated by correlating the differences with the means [11], and the Bradley–Blackwood test assessed equality of means. Sex moderation of the relationship between skinfold and ultrasound measurements was tested using interaction terms in linear regression models. Analyses were performed in Stata 14.0 (Stata Corp., College Station, Texas, USA), with statistical significance set at *p* < 0.05.

## Results

The final sample comprised 146 older adults (118 females and 28 males). Four participants were excluded because they did not have all the SFT measures. Females had higher subcutaneous fat than males at the triceps (skinfold: *p* < 0.001; ultrasound: *p* = 0.003), biceps (skinfold: *p* < 0.001; ultrasound: *p* = 0.046), and subscapular (ultrasound: *p* = 0.048), while age and BMI were similar between sexes. No significant sex moderation was observed for the triceps (β = 0.48; 95% CI −1.44 to 2.41; *p* = 0.623), biceps (β = 0.40; 95% CI −1.25 to 2.05; *p* = 0.631), subscapular (β = 0.68; 95% CI −0.05 to 1.42; *p* = 0.070), or suprailiac sites (β = 1.65; 95% CI −0.68 to 3.98; *p* = 0.165). The mean skinfold thickness values were significantly higher than the corresponding ultrasound measurements across all body sites assessed (*p* < 0.05). The characteristics of the participants are presented in Table 1.

**Table 1:** Participants characteristics.

Variables	Female (*n* = 118)	Male (*n* = 28)	All (*n* = 146)	*p*-value
Age, years	69 (9.0)^a^	71 (5.0)^b^	70 (9.0)^b^	0.254
Body mass index, kg/m²	28.3 (7.7)^a^	28.9 ± 3.5^b^	28.9 ± 4.9^b^	0.624
Skinfold (caliper), mm				
Triceps brachialii	21.2 ± 5.6^b^	13.2 (4.8)^a^	19.8 ± 6.1^b^	<0.001*
Biceps brachii	14.9 (7.2)^a^	10.5 (3.6)^a^	14.5 ± 5.3^b^	<0.001*
Subscapular	21.1 ± 7.3^b^	22.7 ± 6.6^b^	20.9 (10.6)^a^	0.283
Suprailiac	16.3 (7.9)^a^	14.3 (4.8)^a^	16.8 ± 5.9^b^	0.223
Subcutaneous fat thickness (ultrasound), mm				
Triceps brachii	14.1 ± 4.3^b^	9.5 (5.9)^a^	13.6 ± 4.4^b^	0.003*
Biceps brachialii	12.8 (6.6)^a^	10.8 (4.7)^a^	13.0 ± 4.1^b^	0.046*
Subscapular	7.7 ± 1.8^b^	6.6 (1.9)^a^	7.6 ± 1.8^b^	0.048*
Suprailiac	12.7 (6.7)^a^	10.3 (2.1)^a^	12.7 ± 6.1^b^	0.163

Data are presented as mean ± standard deviation for normal ^b^variables and median (interquartile range) for non-normal ^a^variables according to the Shapiro–Wilk test; the Mann–Whitney test was used to compare non-normal variables, and Student’s *T* test for independent samples was used for normal variables; indicates significantly different *p < 0.05.

According to Table 2, the agreement between skinfold caliper and ultrasound measurements was poor at all regions, with the lowest values observed at the triceps (CCC = 0.214) and subscapular (CCC = 0.025) sites. The biceps (CCC = 0.345) and suprailiac (CCC = 0.331) regions showed slightly higher but still poor concordance. Accuracy ranged from low at the subscapular (0.11) to good at the biceps (0.91) and suprailiac (0.81) sites. Systematic bias was evident for the triceps (bias = 0.33) and subscapular (0.88) regions, while the suprailiac site showed no proportional bias (−0.04). The Bradley–Blackwood test confirmed significant differences between methods at all sites (*p* < 0.001). Compared with portable A-mode ultrasound, skinfold calipers consistently overestimated subcutaneous fat across all sites, highest at the subscapular (190.7%) and lowest at the biceps (17.9%), with intermediate values at triceps (56.4%) and suprailiac (50.3%).

**Table 2:** Agreement between subcutaneous fat thickness (portable A-mode ultrasound) and skinfold (caliper) across anatomical sites in older adults (*n* = 146).

Anatomical site	Lin’s CCC	95% CI	Pearson’s r	Accuracy	Mean difference (mm)	Skinfold/ultrasound ratio	95% limits of agreement (mm)	Bias	Bradley–Blackwood *p*-value
Triceps brachii	0.214	0.124–0.303	0.38	0.56	+6.3	1.5	−5.50 to +18.07	0.33	<0.001
Biceps brachii	0.345	0.213–0.477	0.37	0.92	+1.5	1.2	−8.93 to +11.96	0.27	<0.001
Suprailiac	0.331	0.212–0.450	0.41	0.81	+4.1	1.5	−8.74 to +16.96	0.04	<0.001
Subscapular	0.025	0.007–0.043	0.23	0.11	+13.7	2.9	0.06 to +27.39	0.88	<0.001

*CCC* Lin’s concordance correlation coefficient, *95% CI* 95% Confidence Interval.

## Discussion

This study shows low agreement between subcutaneous fat measurements by calipers and portable A-mode ultrasound in older adults, with systematic bias across all sites. Although some regions (biceps, suprailiac) showed reasonable accuracy, skinfolds consistently overestimated fat, particularly at the subscapular site, indicating that the methods are not interchangeable.

Accuracy varied markedly by site, being very low at the subscapular region (0.11) and moderate - good at the biceps (0.91) and suprailiac (0.81). Measurements were closer to perfect agreement at more superficial, anatomically well-defined fat depots. However, higher accuracy did not translate into acceptable overall agreement. This was due to the presence of imprecision and systematic bias, reflected in low CCC values.

Unlike previous studies reporting strong correlations between caliper and ultrasound measurements (*r* = 0.80–0.95) [5–7], this study found lower agreement (*r* = 0.23–0.41). Differences may stem from methodological factors, such as probe frequency, pressure, measurement sites, and the practice of halving caliper values in some studies. Additionally, our sample of older community-dwelling adults contrasts with prior research in younger or athletic populations, where subcutaneous tissue distribution and compressibility differ, likely contributing to weaker correlations in this study.

In our study, skinfold measurements overestimate subcutaneous fat compared to ultrasound, particularly at the subscapular site. Similar site-specific discrepancies have been reported by Müller et al., reflecting differences in skin compressibility, fat distribution, and underlying connective tissue, which likely contribute to variability in the skinfold-to-ultrasound ratios across anatomical sites [5].

Evidence from B-mode ultrasound studies suggests that site-specific discrepancies in subcutaneous fat assessment are not unique to A-mode devices [12]. The subscapular site is particularly challenging for ultrasound assessment due to artefacts from underlying bone, increasing measurement uncertainty [12]. Greater discrepancies at the subscapular site likely reflect anatomical and methodological challenges, including scapular curvature, difficulty in stable probe positioning, and more fibrous, less compressible tissue, which increase variability and overestimation. These factors raise operator dependency and reduce consistency compared with more accessible sites. In contrast, the biceps is less affected by artefacts, although reliability may decline when fat layers are thin [12].

From a clinical perspective, skinfold-based body fat estimates in older adults should be interpreted with caution due to age-related changes affecting accuracy, and portable ultrasound should not be used as a direct substitute without proper validation for individual assessment.

Main limitations include the lack of a gold-standard reference method (e.g., Computed Tomography or Magnetic Resonance Imaging), the operator-dependent nature of A-mode ultrasound, and a predominantly female sample limiting generalizability to males; future studies should include reference imaging and balanced sex distributions. Still, this appears to be the first study comparing subcutaneous fat thickness by portable A-mode ultrasound and skinfold calipers in older adults.

In conclusion, portable A-mode ultrasound and skinfold calipers showed poor concordance and systematic bias across all anatomical sites. Skinfolds overestimated subcutaneous fat, especially at the subscapular region, indicating that the methods are not interchangeable in older adults. Method interchangeability should be avoided in longitudinal monitoring, as switching techniques may create spurious changes; site- and method-specific protocols are needed for clinicians and gerontology researchers assessing subcutaneous fat in older adults.

## Data Availability

The dataset supporting the conclusions of this article is included within the article. Data are available from the corresponding author upon reasonable request.
